# Respiratory weakness after mechanical ventilation is associated with one-year mortality - a prospective study

**DOI:** 10.1186/s13054-016-1418-y

**Published:** 2016-07-31

**Authors:** Clément Medrinal, Guillaume Prieur, Éric Frenoy, Aurora Robledo Quesada, Antoine Poncet, Tristan Bonnevie, Francis-Edouard Gravier, Bouchra Lamia, Olivier Contal

**Affiliations:** 1 Intensive Care Unit Department, Groupe Hospitalier du Havre, Avenue Pierre Mendes France, 76290 Montivilliers, France; 2 Groupe de Recherche sur le Handicap Ventilatoire, UPRES EA 3830, Haute-Normandie Institute of Biomedical Research and Innovation, Rouen University, Rouen, France; 3 Department of Health and Community Medicine, University Hospitals and University of Geneva, Geneva, Switzerland; 4 ADIR Association, Bois Guillaume, France; 5 University of Applied Sciences and Arts Western Switzerland (HES-SO), Lausanne, Switzerland

**Keywords:** Diaphragm, ICU, Maximal inspiratory pressure, Mechanical ventilation, Mortality

## Abstract

**Background:**

Diaphragm dysfunction in mechanically ventilated patients is associated with poor outcome. Maximal inspiratory pressure (MIP) can be used to evaluate inspiratory muscle function. However, it is unclear whether respiratory weakness is independently associated with long-term mortality. The aim of this study was to determine if low MIP is independently associated with one-year mortality.

**Methods:**

We conducted a prospective observational cohort study in an 18-bed ICU. Adults requiring at least 24 hours of mechanical ventilation with scheduled extubation and no evidence of pre-existing muscle weakness underwent MIP evaluation just before extubation. Patients were divided into two groups: low MIP (MIP ≤30 cmH_2_O) and high MIP (MIP >30 cmH_2_O). Mortality was recorded for one year after extubation. For the survival analysis, the effect of low MIP was assessed using the log-rank test. The independent effect of low MIP on post mechanical ventilation mortality was analyzed using a multivariable Cox regression model.

**Results:**

One hundred and twenty-four patients underwent MIP evaluation (median age 66 years (25^th^–75^th^ percentile 56–74), Simplified Acute Physiology Score (SAPS) 2 = 45 (33–57), duration of mechanical ventilation 7 days (4–10)). Fifty-four percent of patients had low MIP. One-year mortality was 31 % (95 % CI 0.21, 0.43) in the low MIP group and 7 % (95 % CI 0.02, 0.16) in the high MIP group. After adjustment for SAPS 2 score, body mass index and duration of mechanical ventilation, low MIP was independently associated with one-year mortality (hazard ratio 4.41, 95 % CI 1.5, 12.9, *p* = 0.007). Extubation failure was also associated with low MIP (relative risk 3.0, 95 % CI 1, -9.6; *p* = 0.03) but tracheostomy and ICU length of stay were not.

**Conclusion:**

Low MIP is frequent in patients on mechanical ventilation and is an independent risk factor for long-term mortality in ICU patients requiring mechanical ventilation. MIP is easily evaluated at the patient’s bedside.

**Trial Registration:**

This study was retrospectively registered in www.clinicaltrials.gov (NCT02363231) in February 2015.

**Electronic supplementary material:**

The online version of this article (doi:10.1186/s13054-016-1418-y) contains supplementary material, which is available to authorized users.

## Background

One of the most common treatments used in intensive care is invasive mechanical ventilation (MV), either via an intubation tube or a tracheostomy. In 2000, an international survey revealed that 39 % of patients admitted to intensive care undergo MV [1]. Under MV, the diaphragm is relaxed. This can cause a specific disorder termed ventilator-induced diaphragmatic dysfunction [2]. The diaphragm atrophies, changes occur in its ultrastructure and contractility is reduced, resulting in a loss of maximal strength [3–5].

Levine et al. showed that 18–69 hours of controlled MV leads to more than 50 % reduction in the cross-sectional area of type I and II diaphragm fibres [3]. This atrophy is the result of a reduction in protein synthesis and acceleration in protein degeneration. After only 6 hours of ventilator-induced diaphragmatic dysfunction, the synthesis of mixed proteins is reduced by up to 30 % and the synthesis of heavy myosin chains is reduced by up to 65 % [6]. Hermans et al. observed negative correlation between the duration of MV and transdiaphragmatic pressure, using magnetic stimulation of the phrenic nerves [7]. Jaber et al. confirmed these results, showing progressive reduction in transdiaphragmatic pressure in ventilated patients, with a 32 % drop in strength after 5 days of MV [4].

Many authors agree that ventilator-induced diaphragmatic dysfunction can increase weaning time and that it is associated with ICU and hospital mortality [4, 8, 9]. It is important to test for ventilator-induced diaphragmatic dysfunction; however, there are few data in the literature on the clinical repercussions of this condition on the overall strength of the inspiratory muscles. De Jonghe et al. found that maximal inspiratory pressure (MIP) below 30 cmH_2_O independently predicts a longer duration of weening from MV [10]. However, more studies have shown that MIP is not sufficiently sensitive to predict extubation failure as it is often multifactorial [11, 12]. Moreover, most studies have only evaluated MIP as a criterion for extubation and the samples included tend to be small [11, 13].

The primary aim of this observational study was to evaluate if a low MIP at the time of liberation following MV was an independent risk factor for mortality. The secondary aim was to evaluate outcomes after extubation.

## Method

This was a prospective observational cohort study conducted in an 18-bed ICU between January 2014 and December 2014. Ethical approval was granted by the French Comité de Protection des Personnes Nord-Ouest 3. The trial is registered as NCT02363231 (www.clinicaltrials.gov).

### Eligibility

Adult patients (age ≥18 years) were eligible for inclusion if they had undergone a minimum of 24 hours of MV and had successfully undergone a spontaneous breathing trial. Patients were excluded in the case of a decision to withhold life-sustaining treatment, degenerative neurological pathology with disabling muscle weakness, chronic loss of autonomy described by the patient’s family (a KATZ score below 6/6 [14]), agitation prior to the evaluation (Ramsay score of 1 or Richmond Agitation-Sedation Scale (RASS) greater than 1), or inability to communicate.

Once the patients were no longer sedated, their state of arousal was evaluated several times per day by the medical and paramedical teams. When they were sufficiently alert and cooperative to respond to instructions (Ramsay score of 2), they underwent a spontaneous breathing trial (inspiratory positive airway pressure of 7 cmH_2_O without expiratory positive airway pressure) for 30 to 120 minutes [15]. If the test failed, the ventilator was returned to the initial settings and the test was repeated at another time. If the spontaneous breathing trial was successful and extubation was planned, the patient was included and underwent an evaluation of MIP.

At inclusion, the following were noted: demographic characteristics, reason for admission, comorbid factors associated with muscle weakness or early loss of functional capacity (chronic respiratory failure, obesity, chronic cardiac failure, cancer, chronic renal failure, diabetes mellitus), and factors relating to the severity of the pathology (Simplified Acute Physiology Score 2 (SAPS2), septic shock, use of corticosteroids or curare and number of days of MV).

Mortality (of any cause) was registered at the follow up one year after successful extubation. Following discharge, patients were contacted every three months by telephone. If the patient had died, the family was asked to specify the date of death. If the family could not be contacted, the mortality status of the patient was obtained by checking local registries.

### Evaluation of MIP

We used an electronic manometer, micro-RPM® (Eolys, PAYS), with a unidirectional valve to measure inspiratory pressure. The manometer was connected to the endotracheal intubation tube with a catheter mount. The back-rest of the patient’s bed was inclined to 45°. Endotracheal suction was carried out to evacuate secretions. Patients were informed that MIP would be evaluated at the residual volume and were instructed accordingly. The patient was disconnected from the ventilator for a minimum of 20 seconds [16]. Three measurements of MIP were carried out and the best was used in the analysis. Finally, we defined low MIP as lower than or equal to 30 cmH_2_O [10, 17].

### Study outcomes

The primary outcome was death during the year following extubation. Secondary outcomes were 30-day rate of mortality, extubation failure, rate of tracheostomy, ICU readmission, non-ICU readmission, correlation between MIP and duration of mechanical ventilation prior to the MIP measure and duration of administration of curare. If the extubation failed, the first MIP carried out was used in the analysis.

### Statistical analysis

Patients’ characteristics were described by frequencies and percentages for categorical parameters and as medians and 25^th^–75^th^ percentiles for continuous parameters. Patients’ baseline characteristics were compared between groups with low and normal MIP using the Student *t* test or the Wilcoxon-Mann-Whitney test, as appropriate, for continuous variables and using the Chi-square test or Fisher exact test, as appropriate, for categorical variables. For the survival analysis, survival curves were estimated using the Kaplan-Meier method and the effect of low MIP was assessed using the log-rank test. Finally, we estimated the effect of low MIP on survival, adjusted for SAPS2, body mass index (BMI) and duration of MV, using a multivariable Cox regression model. We chose to dichotomize the MIP variable (using a clinical cutoff of 30 cmH_2_O) because the survival analysis showed that the relationship between MIP and mortality was not linear.

Statistical analyses were performed using R software (Vienna, Austria, URL http://www.R-project.org/). A two-tailed *P* value of 0.05 was considered significant for all analyses.

## Results

### Patient demographics

**Fig. 1:**
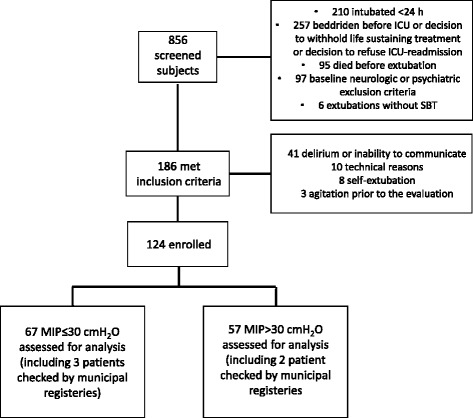
Flow chart. *MIP* maximal inspiratory pressure, *SBT* spontaneous breathing trial

We screened 856 patients and 186 patients fulfilled the inclusion criteria. Of these, 62 had one or more exclusion criteria (e.g. inability to communicate, agitation) or could not undergo MIP evaluation (e.g. self-extubation), as shown in Fig. 1. Therefore, 124 participants were enrolled in the study and underwent MIP evaluation. The characteristics of the 124 participants are presented in Table 1. Briefly, 41 % of the patients were women, median age was 66 years, median BMI was 27.8 Kg/m^2^, median SAPS2 score was 45, median duration of MV was 7 days and median MIP was 29 cmH_2_O.

**Table 1:** Cohort characteristics

	All patients	Low MIP	High MIP	*P* value
	*n* = 124	*n* = 67	*n* = 57	
Female, *n* (%)	51 (41)	30 (45)	21 (37)	0.48
Age, median (25^th^–75^th^ percentile)	66 (56–74)	69 (58–75)	64 (53–70)	0.11
Body mass index (Kg/m^2^), median (25^th^–75^th^ percentile)	27.8 (25–32)	27.4 (24–31)	28.4 (25–33)	0.10
SAPS2 at ICU admission, median (25^th^–75^th^ percentile)	45 (33–57)	46 (31–57)	45 (33–57)	0.46
Admissions to ICU within the last year, *n* (%)	8 (6)	3 (4)	5 (9)	0.33
Main diagnosis				
Pneumonia, *n* (%)	33 (26)	18 (27)	15 (26)	1
Sepsis, *n* (%)	9 (7)	4 (6)	5 (9)	0.73
COPD/asthma exacerbation, *n* (%)	21 (17)	12 (18)	9 (16)	0.81
Cardiac failure, *n* (%)	19 (15)	11 (16)	8 (14)	0.80
Drug overdose/acute mental status change, *n* (%)	16 (13)	8 (12)	8 (14)	0.79
Intra-abdominal sepsis with surgery, *n* (%)	20 (16)	10 (15)	10 (17)	0.80
Trauma, *n* (%)	6 (4)	4 (6)	2 (4)	0.68
Comorbidity				
Chronic pulmonary disease, *n* (%)	28 (22)	18 (27)	10 (18)	0.24
Obesity, *n* (%)	44 (35)	16 (24)	20 (35)	0.17
Chronic cardiac insufficiency, *n* (%)	19 (15)	11 (16)	8 (14)	0.71
Cancer, *n* (%)	19 (15)	13 (19)	6 (11)	0.71
Chronic kidney disease, *n* (%)	20 (16)	8 (12)	12 (21)	0.16
Diabetes mellitus, *n* (%)	30 (24)	12 (18)	18 (31)	0.07
Between admission and awakening				
Septic shock, *n* (%)	61 (49)	33 (49)	28 (49)	0.98
ARDS, *n* (%)	11 (9)	3 (4)	8 (14)	0.11
Renal failure, *n* (%)	38 (31)	19 (28)	19 (33)	0.55
Use of cathecolamines, *n* (%)	64 (52)	35 (52)	29 (51)	0.87
Use of neuromuscular blockers, *n* (%)	76 (61)	40 (60)	36 (63)	0.85
Days on neuromuscular blockers, median (25^th^–75^th^ percentile)	1 (0–3)	1 (0–3)	1(0–2)	0.70
Use of corticosteroids, *n* (%)	34 (27)	21 (31)	13 (23)	0.55
Ventilator use (days), median (25^th^–75^th^ percentile)	7 (4–10)	8 (5–11)	6 (4–8)	0.17

*SAPS2* Simplified Acute Physiology Score 2, *ICU* Intensive Care Unit, *COPD* chronic obstructive pulmonary disease, *ARDS* acute respiratory distress syndrome

### Demographic and clinical factors according to MIP

There were 67 patients (54 %) with low MIP. The demographic characteristics, main diagnosis, severity score and comorbid factors of the patients are presented overall and per MIP group in Table 1. There were no significant differences between MIP groups at admission to ICU.

The median number of days under MV was 8 (5–11) in the low MIP group and 6 (4–8) in the high MIP group; the difference was not statistically significant (*p* = 0.1). There was no correlation between the number of days of MV and MIP (rho = -0.17; *p* = 0.06) and a weak correlation between the number of days under curare and MIP (rho = -0.22; *p* = 0.01). There was no difference between the mean MIP in patients with sepsis and patients without sepsis (respectively, 34.6 ± 18.4 vs. 34.8 ± 20.1 cmH_2_O; difference between means 95 % CI -7, 6.6; *p* = 0.9).

### Effect of MIP on mortality

**Fig. 2:**
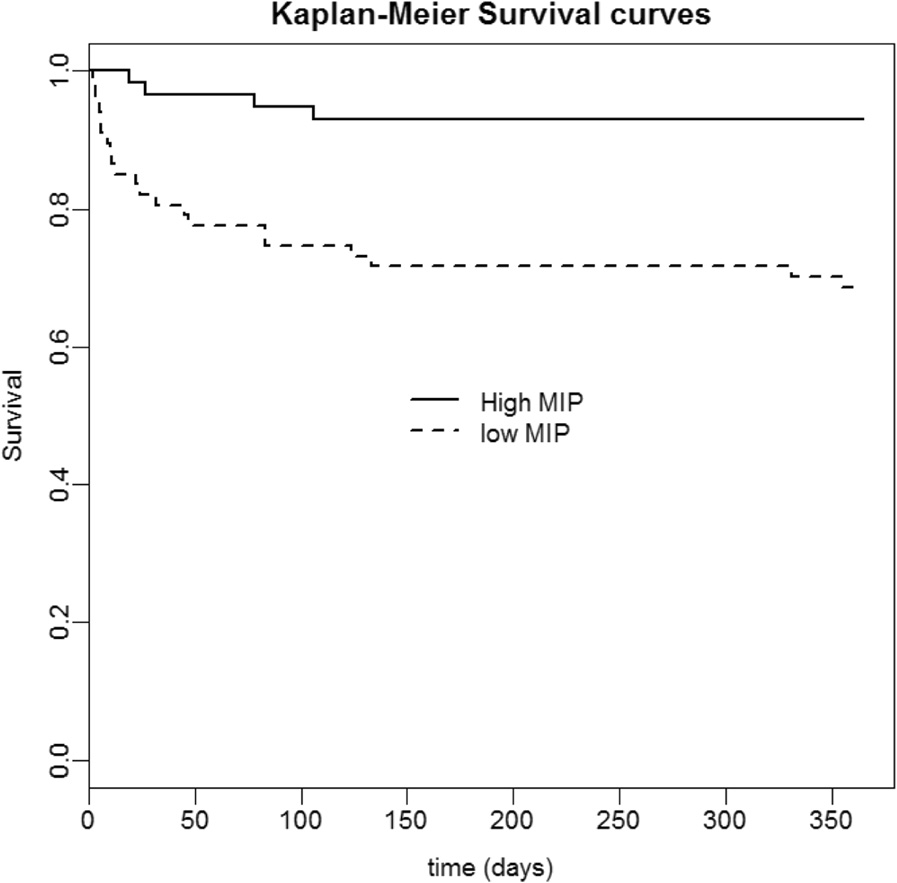
Significant difference between survival curves, based on the log-rank test (*p* < 0.001). Proposition: difference between survival curves (log-rank test (*p* < 0.001)). *MIP* maximal inspiratory pressure

The 365-day mortality was 31 % in the low MIP group and 7 % in the high MIP group. The difference in survival curves in low and high MIP groups was statistically significant (*p* < 0.001, Fig. 2). The effect of low MIP adjusted for SAPS2 score, BMI and duration of MV remained significant in a multivariable Cox regression model (hazard ratio (HR) 4.41, 95 % CI 1.5, 12.9; *p* = 0.007 (Table 2)).

**Table 2:** Multivariable Cox regression model (at one year)

	HR	95 % CI	*P* value
Maximal inspiratory pressure ≤30	4.41	1.51, 12.90	0.007
SAPS2	1.02	1.00, 1.04	0.078
Ventilation (days) >7	1.53	0.68, 3.41	0.30
Body mass index	0.89	0.82, 0.96	0.004

*HR* hazard ratio, *CI* confidence intervals, *SAPS* Simplified Acute Physiology Score

The receiver operator characteristic (ROC) curve for prediction of death by MIP is presented in Additional file 1. The ROC-curve-derived optimal cutoff was 31.5 cmH_2_O with sensitivity of 0.88, specificity of 0.53 and area under the curve of 0.65 (95 % CI 0.54, 0.77) (Additional file 1: Figure S1).

### Clinical outcomes after extubation

Extubation failure and death in ICU were significantly associated with low MIP (Table 3). Tracheostomy and ICU length of stay were not associated with low MIP, nor were ICU or non-ICU readmission.

**Table 3:** Observed outcomes

Outcome after extubation	Low MIP	High MIP	Relative risk for MIP ≤30 cmH_2_O (95 % CI)	*P* value
Extubation failure, *n* (%)	14 (21)	4 (7)	3 (1.1, 9.6)	0.03
Tracheostomy, *n* (%)	1 (1.5)	1 (1.7)	0.8 (0.05, 13.3)	1
Death within 30 days, *n* (%)	12 (18)	2 (3.5)	5 (1.2, 21.9)	0.04
Death in ICU, *n* (%)	10 (15)	1 (1.7)	8.8 (1.1, 64.1)	0.01
Death in hospital, *n* (%)	2 (3)	1 (1.7)	1.7 (0.1-18.2)	1
Death after hospital discharge, *n* (%)	9 (13)	2 (3.5)	3.7 (0.9, 17)	0.06
ICU LOS (days), median (25^th^–75^th^ percentile)	10 (7–16)	9 (5–12)	-	0.14
ICU readmission during the follow-up year, *n* (%)	6 (9)	4 (7)	1.3 (0.4, 2.7)	0.75
Non-ICU readmission during the follow-up year, *n* (%)	6 (9)	10 (17)	0.5 (0.2, 1.3)	0.18

*MIP* maximal inspiratory pressure, *CI* confidence intervals, *ICU* Intensive Care Unit, *LOS* length of stay

## Discussion

The results of this study showed that MIP below 30 cmH_2_O was independently associated with an increased risk of mortality at one year. Moreover, more than 50 % of the patients included had low MIP after mechanical ventilation. Other than a weak correlation with the number of days under curare, there were no other risk factors associated with low MIP.

### MIP, diaphragm dysfunction and mortality

To our knowledge, this is the largest study to evaluate long-term mortality. A cutoff MIP value of 30 cmH_2_O was used in a previous study, but the aim was to evaluate weaning from MV [10]. Several studies have identified a relationship between diaphragmatic dysfunction and mortality [8, 9]. In the present study, we used MIP, which represents the global capacity of the inspiratory muscles (including the diaphragm and accessory inspiratory muscles). Currently, the majority of studies have focused on diaphragmatic dysfunction, with similar results. Supinski et al. observed a 49 % mortality rate in patients with transdiaphragmatic pressure (Pdi) of 10 cmH_2_O (under magnetic stimulation) compared with a rate of 7 % in patients with Pdi above 10 cmH_2_O [9]. Similarly, Demoule et al. observed a higher rate of mortality in the ICU and other hospital wards in patients with diaphragmatic dysfunction evaluated by Pdi during the 24 hours following intubation [8]. However, the evaluation of diaphragmatic dysfunction is difficult. Recently, Supinski et al published an extension of their first study, evaluating an alternative method of assessing inspiratory muscle capacity. They identified correlation between Pdi and MIP, and a relationship between MIP and increased mortality, which corroborates the results of the present study [18].

### Risk factors for weakness and recovery of inspiratory muscles

We chose to evaluate inspiratory muscle capacity just before extubation to isolate the risk factors for mortality relating to hospitalisation in the ICU. The main risk factors for ventilator-induced diaphragmatic dysfunction are duration of MV [7] and sepsis [8, 9]. Surprisingly, in the present study, we did not find a link between MIP, sepsis and the duration of MV. The difference between the results of the present study and that of Demoule and Supinski may be related to the timing of the MIP. They evaluated inspiratory muscle strength during the first 24 hours of MV, whereas we evaluated MIP at the time of extubation, following resolution of the condition that resulted in the need for MV. Recently Dres et al. evaluated the prevalence of diaphragm weakness at the time of liberation from MV [19]. Their univariate analysis showed that only age at admission, and duration of MV were associated with diaphragmatic dysfunction; however, neither of these factors were significant in the multivariate analysis. As in our study, there was no relationship between sepsis and respiratory muscle weakness, suggesting that the risk factors related to diaphragmatic dysfunction at admission can improve with time.

The speed of recovery of the respiratory muscles following weaning from MV is not known. Currently, two studies have evaluated diaphragmatic recovery post MV in rodents. Thomas et al. reported that a 3-hour spontaneous breathing trial improved ventilator-induced diaphragmatic dysfunction after 24 hours of MV and that 4–7 hours of spontaneous breathing trial resulted in a total recovery of the strength and cross-sectional area of type II fibres [20]. Bruells et al. also found a rapid improvement in diaphragm strength in rats 12 hours after cessation of MV [21]. However, total recovery of strength only occurred after 24 hours of spontaneous breathing. No explanation was provided for the difference in these results. Despite these very encouraging results, one of the main limitations of both those studies is that the animals evaluated were healthy. In our study, we did not report the details of the modes of MV but we used a department protocol to quickly shift the patients to pressure support on cessation of neuromuscular blocking agents. Most patients thus underwent pressure support MV, which may have improved their inspiratory muscle strength.

Despite these results, 54 % of the patients in the present study had low MIP, which remains an independent risk factor for mortality. Conversely, we found that BMI was a protective factor against mortality. This paradox, termed the obesity paradox, has already been demonstrated in a large sample of ICU patients [22].

This study has some strong points, including its prospective design and the simple method of evaluation, which allowed us to include a large number of patients. Moreover, this measure is easy to carry out at the patient’s bedside. This is also the first study to have evaluated the clinical consequences of low MIP during the year following discharge from ICU and hospital. However, the study has several limitations. First, it was observational and may suffer from bias inherent to this type of design. More particularly, the small number of events observed limited our multivariate models. The lack of power meant that we could not fully adjust for the effect of comorbid factors (age, sepsis, renal failure, etc.), thus, there may be some residual confounding. However, the most important clinical outcomes were included in the model. Second, we do not have data on the patients’ MIP prior to admission. Third, we did not identify the causes of mortality, and, other than the rate of readmissions, we lack information on the patients’ care pathways following discharge, including any rehabilitation they may have undergone. Last, the use of MIP to evaluate diaphragm dysfunction in the ICU is controversial. Nevertheless, Caruso et al. [16] proposed a standardized method to obtain a reproducible, maximal value of MIP in ventilated patients. We thus followed this method in sufficiently aroused patients (Ramsay score of 2) to ensure maximal participation.

## Conclusion

The results of this study suggest that MIP ≤30 cmH_2_O in patients in the ICU is an independent risk factor for mortality. This measure can easily be carried out at the patient’s bedside. The evaluation of MIP following weaning from mechanical ventilation is very useful clinically. Future studies should confirm these results and analyse the kinetics of recovery from diaphragmatic dysfunction in humans.

## Abbreviations

BMI, body mass index; CI, confidence intervals; HR, hazard ratio; ICU, Intensive Care Unit; MIP, maximal inspiratory pressure; MV, mechanical ventilation; Pdi, transdiaphragmatic pressure; ROC, receiver operating characteristic; SAPS, Simplified Acute Physiology Score

## Additional file

Additional file 1: Figure S1. Receiver operating characteristic (ROC) curve of maximal inspiratory pressure predictive of one-year mortality. (PDF 89 kb)
