# Supplementary figures and images for: Respiratory weakness after mechanical ventilation is associated with one-year mortality - a prospective study
Source: Crit Care. 2016 Jul 31;20:231. doi: 10.1186/s13054-016-1418-y (PMC4967510; doi:10.1186/s13054-016-1418-y)

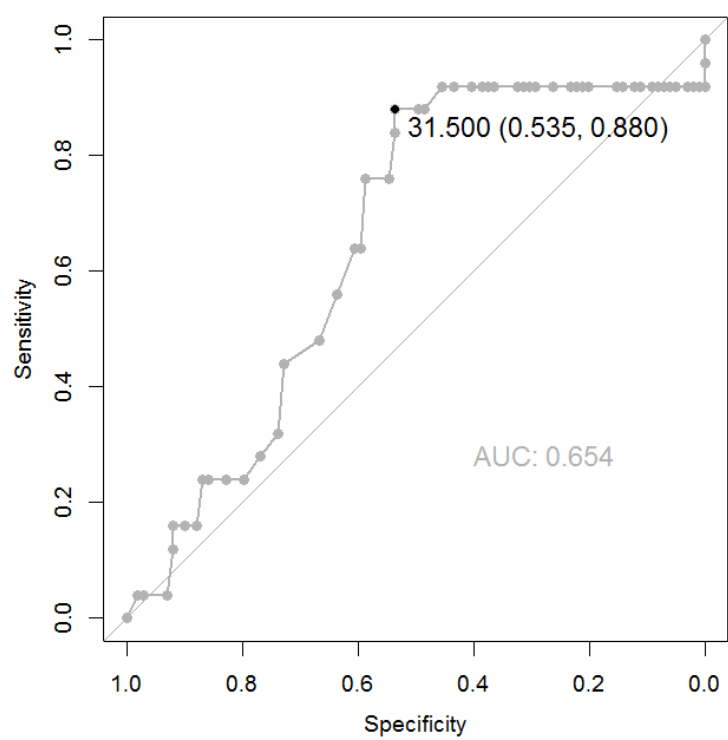

Supplement: Additional file 1: Figure S1. — Receiver operating characteristic (ROC) curve of maximal inspiratory pressure predictive of one-year mortality. (PDF 89 kb) [file 13054_2016_1418_MOESM1_ESM.pdf]
